# Diversification of *Orientia tsutsugamushi* genotypes by intragenic recombination and their potential expansion in endemic areas

**DOI:** 10.1371/journal.pntd.0005408

**Published:** 2017-03-01

**Authors:** Gwanghun Kim, Na-Young Ha, Chan-Ki Min, Hong-Il Kim, Nguyen Thi Hai Yen, Keun-Hwa Lee, Inbo Oh, Jae-Seung Kang, Myung-Sik Choi, Ik-Sang Kim, Nam-Hyuk Cho

**Affiliations:** 1 Department of Microbiology and Immunology, Seoul National University College of Medicine, Seoul, Republic of Korea; 2 Department of Biomedical Sciences, Seoul National University College of Medicine, Seoul, Republic of Korea; 3 Department of Microbiology and Immunology, Jeju National University School of Medicine, Jeju, Republic of Korea; 4 Environmental Health Center, University of Ulsan College of Medicine, Ulsan, Republic of Korea; 5 Department of Microbiology, Inha University School of Medicine, Incheon, Republic of Korea; 6 Institute of Endemic Disease, Seoul National University Medical Research Center and Bundang Hospital, Seoul, Republic of Korea; University of Tennessee, UNITED STATES

## Abstract

**Background:**

Scrub typhus is a mite-borne febrile disease caused by *O*. *tsutsugamushi* infection. Recently, emergence of scrub typhus has attracted considerable attention in several endemic countries in Asia and the western Pacific. In addition, the antigenic diversity of the intracellular pathogen has been a serious obstacle for developing effective diagnostics and vaccine.

**Methodology/Principal findings:**

To understand the evolutionary pathway of genotypic diversification of *O*. *tsutsugamushi* and the environmental factors associated with the epidemiological features of scrub typhus, we analyzed sequence data, including spatiotemporal information, of the *tsa56* gene encoding a major outer membrane protein responsible for antigenic variation. A total of 324 *tsa56* sequences covering more than 85% of its open reading frame were analyzed and classified into 17 genotypes based on phylogenetic relationship. Extensive sequence analysis of *tsa56* genes using diverse informatics tools revealed multiple intragenic recombination events, as well as a substantially higher mutation rate than other house-keeping genes. This suggests that genetic diversification occurred via frequent point mutations and subsequent genetic recombination. Interestingly, more diverse bacterial genotypes and dominant vector species prevail in Taiwan compared to other endemic regions. Furthermore, the co-presence of identical and sub-identical clones of *tsa56* gene in geographically distant areas implies potential spread of *O*. *tsutsugamushi* genotypes.

**Conclusions/Significance:**

Fluctuation and diversification of vector species harboring *O*. *tsutsugamushi* in local endemic areas may facilitate genetic recombination among diverse genotypes. Therefore, careful monitoring of dominant vector species, as well as the prevalence of *O*. *tsutsugamushi* genotypes may be advisable to enable proper anticipation of epidemiological changes of scrub typhus.

## Author summary

Scrub typhus, caused by *Orientia tsutsugamushi* infection, is a mite-borne febrile illness endemic in the Asia-Pacific region. Recent emergence and continuous local outbreaks in many of the endemic countries make it a serious public health issue. In addition, the antigenic diversity of the *tsa56* gene, encoding a major outer membrane protein, hampers the development of effective diagnostics and vaccine. Here, we extensively analyzed *tsa56* sequences and their spatiotemporal information to elucidate the evolutionary pathway of genotypic diversification, as well as the environmental basis associated with the epidemiological changes of scrub typhus. Based on various informatics analyses, we found that genetic diversification of *tsa56* might have been attained via frequent point mutations and subsequent genetic recombination among diverse genotypes. Prevalence of numerous bacterial genotypes and dominant vector species in Taiwan also suggest that the subtropical area located at the center of endemicity, may serve as a local mixing ground for genotype diversification. In addition, detection of identical and sub-identical clones of *tsa56* genes in geographically distant countries indicates a potential spreading of bacterial genotypes. Continuous monitoring of dominant vector species and the associated *O*. *tsutsugamushi* genotypes might be required for developing better diagnostics and an effective vaccine for scrub typhus.

## Introduction

Scrub typhus is an acute febrile illness caused by *Orientia tsutsugamushi* infection. The bacterium is an obligate intracellular pathogen maintained through transovarian and transtadial transmission in trombiculid mites that serve as vectors for the infectious disease [1,2]. The disease is endemic in Asia and the western Pacific area including northern Australia. The first description of a febrile disease thought to be scrub typhus, along with the morphology of the vector mites, appeared in Chinese literature in 313 A.D. [3]. Therefore, it seems to an ancient infectious disease that has long been confined to its endemic area, although several cases of suspected scrub typhus have been reported outside of the endemic region [4–7].

**Fig 1:**
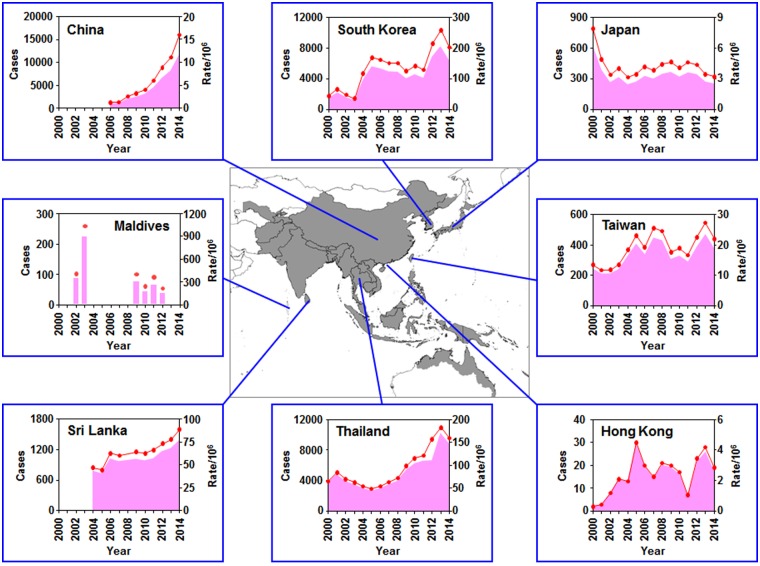
Epidemiological trends of scrub typhus incidence in several endemic countries from 2000 to 2014. Regional map shows the distribution of scrub typhus (gray area) and the annual incidence of several endemic countries during 2000 ~ 2014 are presented. The graphs are based on the data summarized in S1 Table. Red line: reported cases, pink area: incidence rate/10^6^.

Global incidence of scrub typhus across the whole endemic region has been poorly defined due to the limited epidemiological data in many of the endemic countries. Nevertheless, it has been estimated that more than a million cases occur annually and a billion people are at risk [8]. In addition, there has been a rapid increase in scrub typhus cases, as well as sporadic local outbreaks during the last decade [9–13], making it a serious public health issue in the endemic area. Recent epidemiological data available in various resources (S1 Table), clearly demonstrates the gradual emergence of scrub typhus in several endemic countries (Fig 1). Even though the increasing number of reported cases of scrub typhus might be partly due to increased awareness and better surveillance systems in the developing countries [2,9], environmental change and human activity might be important factors contributing to the emerging trend [14–17]. Given that vector mites maintain the intracellular pathogen, ecological changes of the vector species in local endemic regions could be the primary cause of the emergence of scrub typhus, as recently observed in South Korea [15,16]. However, the distribution of mite species associated with scrub typhus in the whole endemic region has been poorly monitored and the currently available vector map issued by the World Health Organization (WHO) is based on data before 1974 [18–20].

Another critical issue of scrub typhus is the apparent antigenic diversity of *O*. *tsutsugamushi* throughout the region of endemicity [1]. The antigenic heterogeneity has been a serious obstacle for developing effective diagnostic methods, as well as a universal scrub typhus vaccine [1,2]. Historically, the antigenic variation of *O*. *tsutsugamushi* was characterized by several serological techniques using whole bacterial antigens, such as complement fixation and immunofluorescence assay. Based on serological analyses, the bacterial pathogen has been classified into various strains, including Karp, Gilliam, and Kato prototypes [21]. The defined “serotypes” have been changed into “genotypes” since a 56 kDa type-specific antigen, *tsa56*, occupying approximately 20% of whole bacterial proteome [22], was identified to be a major bacterial surface antigen reactive to strain-specific antibodies [23]. Genetic analysis of the *tsa56* gene has provided the most useful standard to differentiate the genotypes of *O*. *tsutsugamushi* [22,24,25] and a growing number of *tsa56* sequences has been deposited in the international nucleotide database [26]. As the number of *tsa56* sequences increases, more diverse genotypes of *O*. *tsutsugamushi* have been identified [1,27]. Nevertheless, the question of how the genetic diversity of *tsa56* gene has evolved remains unsolved. In addition, the potential relationship between genotype variation and epidemiological changes has been poorly assessed in the global level.

In order to understand the evolutionary pathway of genotypic diversification of *O*. *tsutsugamushi*, as well as the environmental basis associated with the epidemiological changes of scrub typhus, we collected and analyzed the data of *tsa56* genes, including genetic sequences and their spatiotemporal information. We also searched and reviewed references containing information on the *Leptotrombidium* species, the primary vectors of scrub typhus, to update the vector map and examine ecological changes of the “natural host” of *O*. *tsutsugamushi* in the whole endemic region. The systemic analysis of genotype diversity and geographical distribution of the vector hosts may not only provide valuable insight into the factors affecting epidemiological changes, but also enhance our current knowledge required for developing better diagnostics and an effective vaccine for scrub typhus.

## Methods

### Data collection

Annual incidences of scrub typhus in endemic countries were collected from various references and resources (S1 Table). The number of scrub typhus cases reported in each country has been based on different diagnosis standards. The criteria for confirmed cases of scrub typhus in China, Japan, and Taiwan include clinical manifestations, and one of the following laboratory diagnosis criteria: serological analysis such as indirect immunofluorescence assay, detection of *O*. *tsutsugamushi* DNA by PCR, or isolation of the pathogen from clinical specimens. Data from other countries include both clinically suspected cases, which are solely based on epidemiological exposure histories and clinical symptoms, and confirmed cases by laboratory diagnosis as described above.

Nucleotide sequences encoding the *tsa56* gene were collected from the National Center for Biotechnology Information (NCBI, http://www.ncbi.nlm.nih.gov/). As of Dec. 31. 2015, 1,030 nucleotide sequences have been deposited in the sequence databases. Among them, we selected and analyzed 324 nucleotide sequences that covered at least 85% of the open reading frame. Other information of the selected *tsa56* genes, such as isolation host, year of isolation, and isolated location, were also retrieved from the database or manually collected from the references citing the sequences, and summarized in S2 Table. Based on the phylogenetic analyses described below, we re-annotated the genotypes of the 324 sequences and selected a representative proto-genotype sequence for each genotype. The proto-genotype sequences were selected if genomic information was available or its genome sequencing was underway in Bioproject (http://www.ncbi.nlm.nih.gov/bioproject/). If there was no genomic information available, the proto-genotype was annotated to a sequence which was firstly reported among the genotype members.

We also searched and reviewed literature for epidemiological data on the distribution of nine *Leptotrombidium* mite species, the primary vectors responsible for the transmission of scrub typhus, to construct and update the vector map. The spatiotemporal information of the mite species is summarized in S3 Table. The geographical distribution of the vector species was based on locations described in literature using the QGIS program (http://qgis.org/) and map dataset available in Natural Earth (http://www.naturalearthdata.com/). If a geographical reference did not include specific decimal latitude and longitude information, we used area information (generally province or county level) described in literature.

### Sequence analysis and phylogenetic classification of genotypes

The sequence data of *tsa56* genes were translated and analyzed using the MEGA6 software [28]. Since the length of nucleotide and amino acid sequences were quite variable, we first aligned the amino acid sequences using the MAFFT algorithm (v.7.) with the E-INS-i option [29], manually checked the aligned sequences, and trimmed them to select the sequence region shared by all 324 genes [30]. The lengths of the selected amino acid sequences range from 417 (1251 bases) to 467 (1401 bases) (S2 Table), and cover the majority of the extracellular region and excludes the signal peptide of the TSA56 protein. Among the 324 genes analyzed, 156 were found to share identical nucleotide sequences in at least two genes. Therefore, we annotated 206 sequence IDs to the gene set (S2 Table) and analyzed them for genetic relationships. Phylogenetic analysis of the aligned nucleotide sequences was performed using the MAFFT algorithm and the Randomized Axelerated Maximum Likelihood (RAxML) method as implemented in SeaView software (v. 4.5.1) [31]. Shimodaira-Hasegawa-like (SH-like) test [32] was computed to measure the statistical support of ML tree, as implemented in RAxML. The values for SH-like branch support are presented at the nodes on the phylogenetic tree and values above 0.9 were considered as significant phylogenetic support. Pairwise identity and similarity matrices of amino acid sequences were constructed by the MatGAT2.1 program [33], which aligned the sequences using the BLOSUM62 matrix.

### Estimation of genetic recombination

Intragenic recombination was screened within the aligned sequences using the Genetic Algorithm Recombination Detection (GARD) method [34] implemented in Datamonkey server [35]. This program identifies the number and location of breakpoints and sequences involved in putative recombination events. In addition, seven methods implemented in Recombination Detection Program (RDP4) suite [36] were also applied to detect potential recombinant sequences, parental sequences, and recombination breaking points: 3Seq [37], Bootscan [38], Chimaera [39], GENECONV [40], MaxChi [41], RDP [36], and SisScan [42]. Analyses were performed with default settings for the detection methods and each potential event was considered significant when a support *p* value was less than 0.05 by more than six detection methods. The breakpoints and recombinant sequences inferred for every potential event were manually checked and adjusted using the phylogenetic and recombination signal analyses available in RDP4 suite. A similarity plot of *tsa56* nucleotide sequences displaying the extent of genetic diversity between the significantly related genotypes was generated using a window of 200 nucleotides and a step of 20 nucleotides.

In order to estimate and compare the degree of genetic diversity of *tsa56* genes with other *O*. *tsutsugamushi* genes, we collected 53 bacterial genes including the *tsa56* gene from two complete genome sequences (Boryong [43] and Ikeda strain [44]) and seven draft genomic contigs (strain:Bioproject accession no.; Gilliam:PRJNA212442, Karp:PRJNA212456, Kato:PRJNA212440, TA716: PRJNA212457, TA763:PRJNA212454, UT76:PRJNA212456, UT144:PRJNA232539) available in Bioproject (http://www.ncbi.nlm.nih.gov/bioproject/). All selected 53 genes are present in at least eight genomes. Nucleotide diversity (π, the average number of nucleotide differences per site), mutation rate (θ, Watterson’s mutation parameter), and recombination parameter (ρ) of the gene sets were estimated by LDHat package implemented in RDP4 suite. The number of non-synonymous (*Ka*) and synonymous (*Ks*) substitutions for each gene locus were calculated by using the SeqinR package in the R-project. We also identified genotype-specific insertions/deletions (Indels) in *tsa56* genes by manual inspection of 206 aligned sequences and summarize them in S4 Table.

## Results

### Phylogenetic analysis of *tsa56* genes

**Fig 2:**
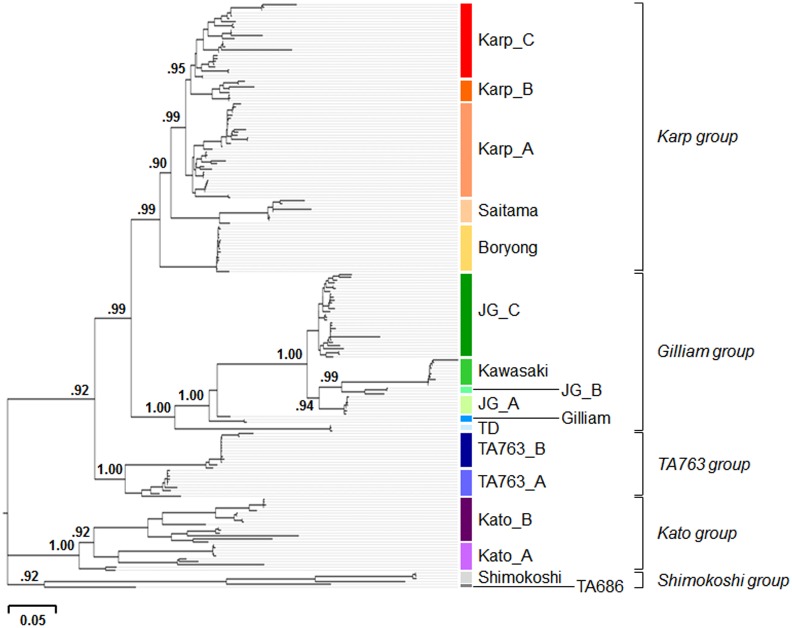
Phylogenetic analysis of 206 *tsa56* genes and their classification into genotypes and genogroups. Phylogenetic relationships of 206 complete or nearly complete *tsa56* genes (listed in S2 Table) covering more than 85% of coding sequences are presented. 17 genotypes were defined based on the branching supporting values (SH-like value ≥ 0.90) and the relative branch length from a node. They were further classified into 5 genogroups (Karp, Gilliam, TA763, Kato, and Shimokoshi) based on phylogenetic distances.

**Fig 3:**
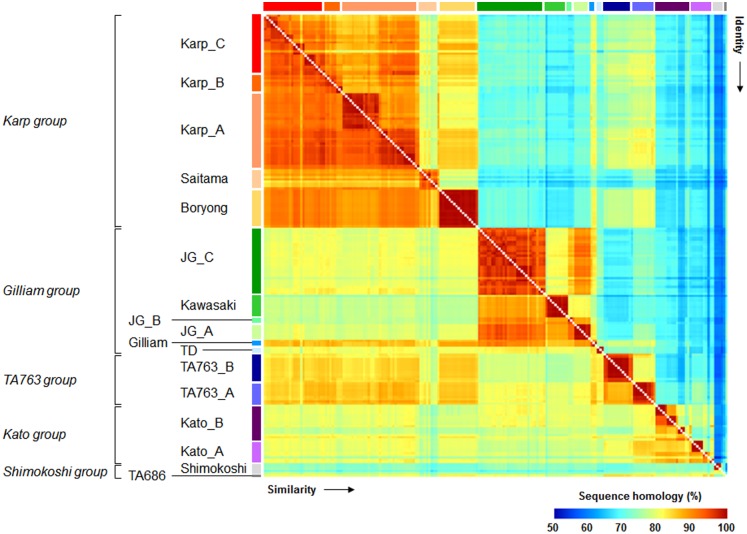
Similarity and identity matrix for amino acid sequences of 206 TSA56 proteins. Pairwise identity (upper triangle) and similarity (lower triangle) matrix of TSA56 sequences were constructed using the MatGAT2.1 program. The order of TSA56 sequences is the same as in Fig 2. Raw data values are presented in S5 Table.

Phylogenetic analysis was performed using 206 unique *tsa56* nucleotide sequences (S2 Table) and defined the genetic clusters based on branching supporting values (SH-like value ≥ 0.90) and the relative branch length from a node. At least 17 genotypes were defined by the phylogenetic analysis of the nucleotide sequences and named after the prototype strains (Fig 2). Based on the phylogenetic distances, 17 genotypes were further classified into 5 groups: Karp, Gilliam, TA763, Kato, and Shimokoshi. When we compared the 206 genes using protein sequences, the ranges of sequence similarity and identity in the amino acid level further support the grouping of genotypes (Fig 3 and S5 Table). Within each genotype, minimum similarity and identity among the gene members are generally over 80.0% and 70.0%, respectively, with the exception of the Shimokoshi genotype (min. similarity: 79.0%, min. identity: 68.2%). Among the gene members within a group, minimum similarity and identity are further reduced to 73.0% and 60.1%, respectively, as observed in the Shimokoshi group. Among all the collected genes, the minimum similarity (66.2%) was observed between members of Saitama and Shimokoshi genotypes and the minimum identity (52.9%) was detected between members of Kato_A and Shimokoshi genotypes. It is worth noting that gene members in the Gilliam genotype and in the TA763 group show relatively higher similarity to those of Karp group (Fig 3), although they are phylogenetically distant.

### Detection of genetic recombination in *tsa56* genes

**Fig 4:**
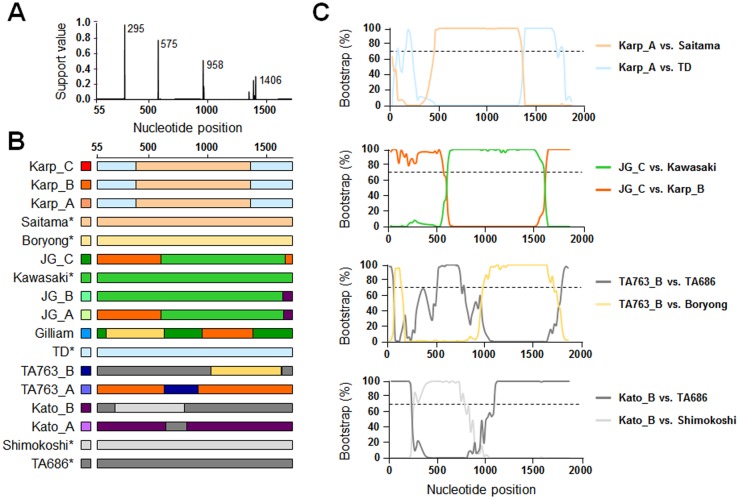
Detection of intragenic recombination in *tsa56* genes using 17 proto-genotype sequences. **A.** Recombination breakpoints within *tsa56* sequences were detected using the GARD program. Support probabilities for inferred recombination break-points are shown on the left side of the breakpoint plots. **B.** Intragenic recombination events in *tsa56* sequences found using the RDP suite. The schematic diagrams of indicated genotypes present the putative major and minor parent sequences of each genotype (by color codes) and the location of predicted breakpoints. Detailed information of the recombination events predicted by the RDP suite is summarized in S6 Table. The genotypes without significant recombination are marked with (*). **C.** Representative BOOTSCAN evidence for recombination origin on the basis of pairwise distance, modeled with a window size 200 nt, step size 20 nt, and 100 Bootstrap replicates. The threshold of significance for the analysis was set as 70% bootstrapping value (dash line).

**Fig 5:**
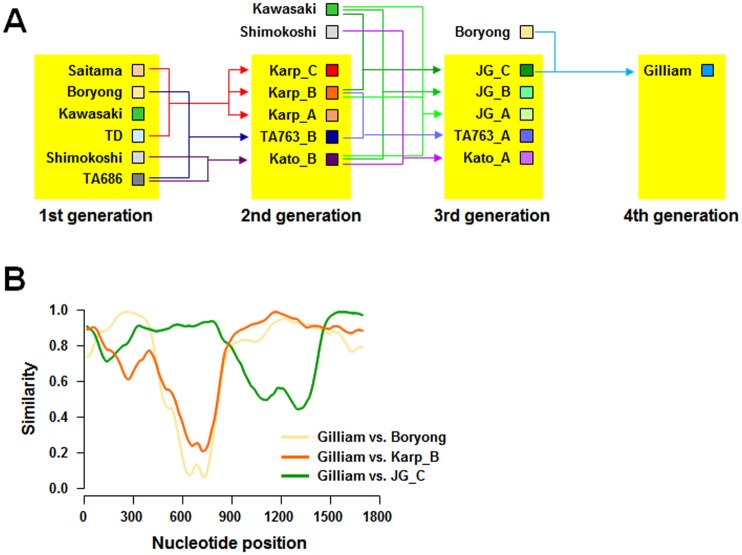
Hierarchical relationship of 17 proto-genotype sequences. **A.** Based on the recombination events and the parental origins of the *tsa56* sequences shown in Fig 4, the hierarchical relationship of 17 genotypes was estimated and is presented as sequential generations. **B.** Recombination events in Gilliam genotype sequence, the last generation in A, were analyzed by similarity plot on the basis of pairwise comparison with Boryong, Karp_B, and JG_C genotypes as parental sequences (scanned with a window size 200 and step size 20 nt).

Since we observed a relative conservation of amino acid sequences among the genotype members belonging to phylogenetically distant groups, such as Gillam and TA763 members with Karp members, and it has long been proposed that genetic recombination might be a driving force for generating genetic diversity of the intracellular pathogen [22,27,43,45–47], we examined the genetic recombination of 17 prototype sequences of *tsa56* using several different recombination detection programs. First, the evidence for recombination in the aligned 17 proto-genotype sequences was tested by the GARD method using the Datamonkey web server (http://www.datamonkey.org) [48]. This method detected evidence for recombination at multiple breakpoints predicted at nucleotide positions 295, 575, 958, and 1406 (*p* < 0.01) (Fig 4A). Detection of potential recombinant sequences, identification of potential parental sequences, and localization of possible recombination break points were further determined using the 3Seq [37], Bootscan [38], Chimaera [39], GENECONV [40], MaxChi [41], RDP [36], and SisScan [42] methods embedded in RDP suite [49]. As shown in Fig 4B and S6 Table, significant recombination events were detected in 11 proto-genotypes with a high degree of confidence (*p* < 0.05 for at least six out of seven recombination detection programs), but not in Saitama, Boryong, Kawasaki, TD, Shimokochi, and TA686 genotypes. Among the 11 proto-genotypes, 9 genotypes showed a single recombination event, whereas multiple recombination events were predicted in 2 genotypes, JG_A and Gilliam. The major parents, minor parents, and break points with statistical significance (*p* < 0.05) confirmed by MaxChi and BOOTSCAN programs are summarized in S6 Table. Fig 4C shows four representative recombination events observed in Karp_A, JG_C, TA763, and Kato_B genotypes. These results suggest that 6 genotypes, Saitama, Boryong, Kawasaki, TD, Shimokoshi, and TA686, may be the ancestral parents of the 11 recombinant genotypes. Based on the number of recombination events observed in the diverse genotypes, we speculate that the recombinant genotypes might have been generated by sequential recombination events among the parental genotypes (Fig 5). The 6 genotypes which lack any evidence of recombination, might be the first generation that contributed to the second generation (Karp_C, Karp_B, Karp_A, TA763_B, and Kato_B). Three members (Kawasaki, Shimokoshi, and Boryong) of the first generation also contributed to the third and fourth generations. Gilliam genotype, the sole member of the fourth generation, seems to have been generated by recombination between Boryong, Karp_B, and JG_C genotypes (Fig 5B). These results suggest that the genotype diversification of *O*. *tsutsugamushi* may be an ongoing process driven by continuous recombination events among preexisting genotypes.

**Fig 6:**
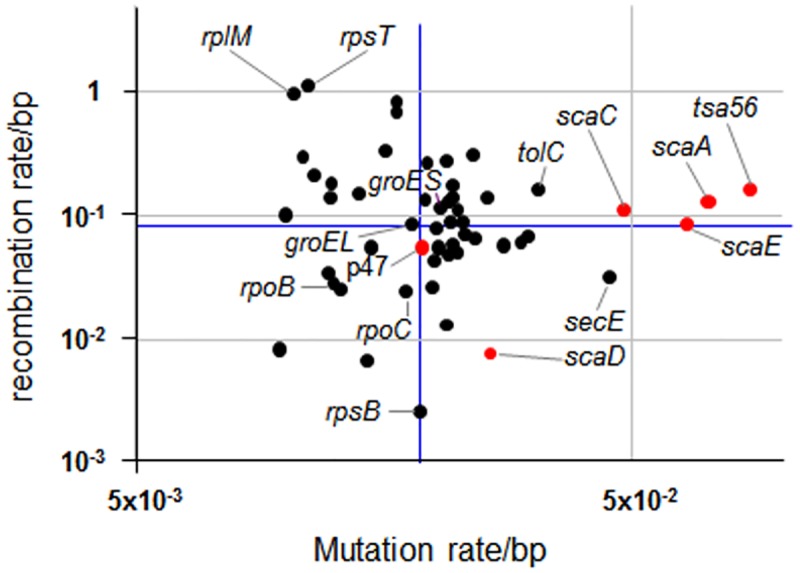
Estimation of recombination and mutation rates of *O*. *tsutsugamushi* genes. Recombination and mutation rate per site of 53 genes were calculated by LDhat installed in RDP program. Sequences of 53 gene sets were extracted from nine *O*. *tsutsugamushi* genomes available in NCBI database. Average recombination rate per base pair (ρ/bp) of 53 gene sets is 0.083 and average mutation rate per base pair (θ/bp) is 0.020 (blue lines). Genes encoding known membrane proteins are indicated as red dots. Detailed information of the gene sets is presented in S7 Table.

Genetic diversification of a bacterial gene can be attributed to point mutations as well as genetic recombination. Therefore, we analyzed the relative contribution of recombination and point mutation to the diversification of *tsa56* genes and compared these results to those of 52 other *O*. *tsutsugamushi* genes collected from nine genomes that are completely sequenced or undergoing sequencing (Fig 6 and S7 Table). Average recombination rate per base pair (ρ/bp) of the 53 genes is 0.083, with a range of 0.003 (*rpsB*) to 1.131 (*rpsT*), and average mutation rate per base pair (θ/bp) is 0.020, with a range of 0.010 (*trmU*) to 0.086 (*tsa56*). The detecting per site ρ/θ value for the overall gene sets is 4.071, suggesting that recombination occurred more frequently than point mutation. Interestingly, the mutation rates of *tsa56* and *sca* family genes, encoding outer membrane proteins [50], are generally higher than other house-keeping genes, even though their recombination rates were near average value. When we recalculated per site recombination and mutation rates using 206 unique *tsa56* genes, ρ/bp and θ/bp are 0.041 and 0.050, respectively, indicating that mutation rate is slightly higher than recombination rate (per site ρ/θ value = 0.812, S7 Table). These results suggest that genetic diversification of the major outer membrane protein, TSA56, might be driven by recombination as well as frequent point mutation. Based on the *Ka*/*Ks* ratio, an indicator of selective pressure acting on a protein-coding region, most *O*. *tsutsugamushi* genes, including *tsa56*, predominantly evolve by purifying selection with the exception of *rplM*, *scaA*, and *scaE*, which may evolve under positive selection.

In addition to the point mutations, Indels of nucleotide sequences have often been observed in Rickettsial genes [51,52] and may also contribute to the genotypic diversification of *Orientia*. Extensive analysis of the aligned 206 *tsa56* genes revealed 4,771 Indels at over 108 sites, especially in regions encoding variable domains [22]. However, only a few of them are consistently detected in a specific set of genotype sequences (S4 Table), indicating that, although they contributed to the diversification of *tsa56* sequences, only fraction of Indels are conserved in a specific genotype.

### Geographical distribution of *O*. *tsutsugamushi* genotypes

**Fig 7:**
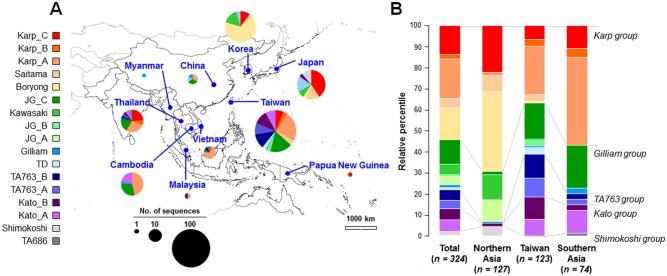
Geographical distribution of *O*. *tsutsugamushi* genotypes in endemic countries. **A.** The relative proportion of each genotype reported in the indicated endemic country is presented and the pie size is proportional to the number of sequences reported. The number of *tsa56* sequences used for each country is as follows: Taiwan (123), Korea (69), Japan (52), Thailand (29), Cambodia (28), China (9), Malaysia (2), Myanmar (1), and Papua New Guinea (1). **B.** Relative proportion of each genotype reported from Taiwan, northern endemic area (Korea, Japan, and China), and southern countries (Thailand, Cambodia, Vietnam, Malaysia, Myanmar, and Papua New Guinea) is presented. The number of sequences from each group is indicated below the bar graphs.

The geographical distribution of 324 *tsa56* genes is presented in Fig 7 and S2 Table. The Karp group includes the largest number of isolates (175 genes) and genotypes in this group are found in most endemic countries. Among the genotype members in the Karp group, each country has a specific predominant genotype, such as Boryong in South Korea, Karp_C in Japan, and Karp_A in Taiwan and Cambodia. Genotype members of the Gilliam group (78 isolates) are also quite prevalent in the endemic countries. Among the genotype members in this group, the Kawasaki genotype is prevalent in South Korea and JG_C genotype is primarily found in Taiwan, Thailand, and Cambodia. The isolates belonging to the TA763 and Kato groups are mainly reported in Taiwan, but rarely in South Korea and China. The highly divergent Shimokoshi genotype is only reported in Japan. Even though the number of sequences isolated from each country is quite varied, the genotype diversity found in Taiwan located in the middle of endemic area of scrub typhus, is particularly notable. 14 genotypes out of 17 are found in Taiwan, compared to 5 genotypes in South Korea. When we compared the diversity and relative proportion of genotypes in Taiwan with those of the northern endemic area (China, Japan, and Korea) or southern endemic countries (Cambodia, Malaysia, Myanmar, Papua New Guinea, Thailand, and Vietnam), it is clear that not only the diversity, but also the relative proportion of each genotype in Taiwan are quite distinct from other endemic regions (Fig 7B). Prevalence of more divergent genotypes in a certain central locality than in countries at the boundary of the endemic region suggests that Taiwan might serve as a mixing ground for the diverse genotypes.

### Distribution of *Leptotrombidium* vector species

**Fig 8:**
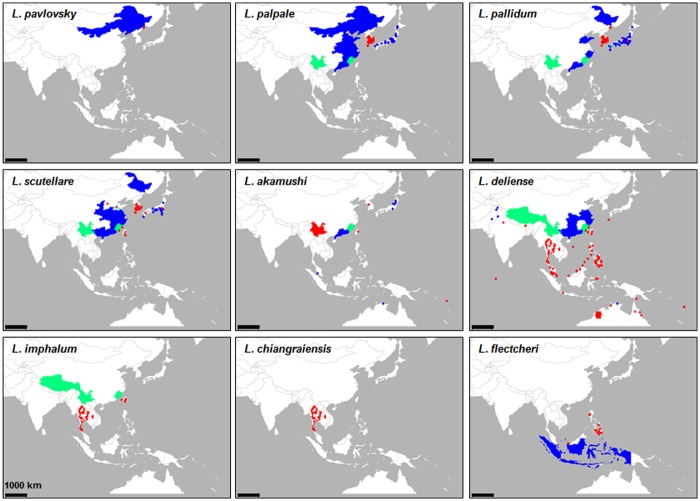
Geographical distribution of nine *Leptotrombidium* species, the major vectors of scrub typhus. Geographical distributions of nine representative *Leptotrombidium* species mediating scrub typhus are presented. If the collection sites of vector identification were specified with coordinates, they are indicated as red dots, otherwise the collection sites were indicated at province or county level as colored area. Blue: collected before 1974, red: collected after 1974, green: collected before and after 1974. Detailed information is available in S3 Table.

The distribution of vector mites is the primary factor affecting the epidemiological features of scrub typhus. However, the currently available vector map, published by the World Health Organization in 1989 [18], is primarily based on data before 1974 [1,3,19,20]. Therefore, we searched references and updated the geographical distribution of the *Leptotrombidium* species, the main vectors of scrub typhus (Fig 8 and S3 Table).

Three major *Leptotrombidium* species, *L*. *palpale*, *L*. *pallidum*, and *L*. *scutellare*, have been found in the northeastern area of endemicity [15,18,53–55]. In particular, *L*. *scutellare* has recently become the primary vector in northern China [56,57], South Korea [15], and Japan [53]. It is also notable that *L*. *pavlovskyi*, the primary vector responsible for scrub typhus in the Primorye region of Russia during the 1960s, was also prevalent in the 1990s, although *O*. *tsutsugamushi* could not be isolated from the vector species [58]. *L*. *delicense* is the primary vector in the southern parts of the endemic region, ranging from southern China to the north, Pakistan to the west, and northern Australia and western Pacific islands to the south. A number of studies have also reported the presence of *L*. *delicense* in south-eastern countries of the endemic region since 1974 (S3 Table). It appears that two *Leptotrombidium* species, *L*. *scutellare* in the northern part and *L*. *delicense* in the southern area of endemicity, might be the primary vectors for current scrub typhus. However, there is local variation of major vectors such as *L*. *imphalum* in eastern Taiwan [59], *L*. *chiangraiensis* and *L*. *imphalum* in northern Thailand [60]. In addition, *L*. *scutellare* has been recently reported to have expanded both northward to mainland China [56,57] and South Korea [15], and the southward to southern China [55] and Taiwan [59,61]. As a result, the southern provinces of mainland China and Taiwan have become mixing grounds for the two primary vector species, *L*. *scutellare* and *L*. *delicense*. It is also notable that *L*. *imphalum*, the primary vector in northern Thailand [60], has also been reported as the dominant mite in a local area of Taiwan [59], such that diverse vector species prevail in islands of Taiwan.

### Presence of identical and sub-identical *tsa56* genes in geographically distant countries

**Fig 9:**
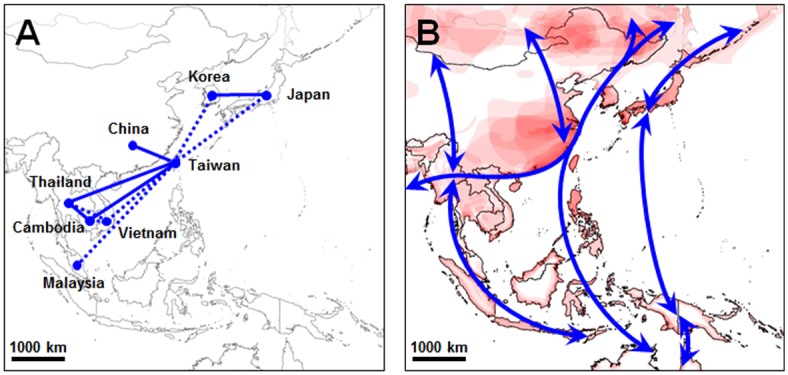
Potential expansion of *O*. *tsutsugamushi* clones throughout the endemic region. **A.** Linkage map indicates the presence of identical (solid line) or sub-identical (one or two base difference, dotted line) *tsa56* genes in geographically distant endemic countries. **B.** The East Asia/Australasia major flyway of migratory birds is presented as blue lines. The primary habitats of 21 key bird species in the flyway are overlaid on the regional map (Data available at http://www.eaaflyway.net/).

As mentioned above, there are 38 sets of identical sequences, including 156 sequences, among the selected 324 *tsa56* genes (S2 Table). Interestingly, among the identical sequence sets, 10 sets of genes include sequences from two different countries (S8 Table), whereas 28 sets were only reported within a single country. For example, the same sequences belonging to Karp_C, Kawasaki, and JG_A have been reported from South Korea and Japan, even though the isolated years are different. Identical sequences in the Karp_A genotype have also been found in Taiwan and Thailand. In addition, we detected sequence pairs, showing only one or two base sequence differences (S8 Table), originated from two different countries, such as Boryong genotype from Korea and Taiwan. The presence of identical or sub-identical (1 ~ 2 different bases) *tsa56* genes in geographically distant countries implies a potential migration or expansion of the bacterial clones, even though it needs to be confirmed whether other bacterial genes and/or the whole genomic sequences of the isolates are also identical. It is also intriguing that Taiwan emerged as a central node connected to northern countries (China, Japan, and Korea), as well as southern countries (Cambodia, Malaysia, and Thailand) when we linked the countries where the identical and sub-identical sequences have been reported (Fig 9A). Considering that prominent diversity in bacterial genotypes and mite vector species have been observed in Taiwan and its central location in the endemic area of scrub typhus, the subtropical region may potentially serve as a hub point mediating migration or expansion of vector mites, thereby contributing to the spread of diverse genotypes and their recombination.

## Discussion

The *O*. *tsutsugamushi* genome is quite unique in that up to 40% of its genome contains dispersed repeat sequences, primarily composed of mobile genetic elements including conjugative transfer (*tra*) components of a type IV secretion system and transposases [43]. Previously, sex pili-like cell surface appendages for conjugal DNA transfer were observed in *Rickettsia belli*, which encodes a *tra* cluster phylogenetically close to those of *O*. *tsutsugamushi* [45,62]. It is possible that genetic recombination occurs via a similar mechanism among *O*. *tsutsugamushi*. Coinfection of multiple genotypes in a single host might facilitate the genetic recombination among different genotypes. Indeed, mixed infection of multiple genotypes has often been reported in the vector mites [63,64] and human patients [46,47,65]. Given that humans are dead-end hosts, recombination between different strains more likely occurs in the mite vectors or in the rodent reservoirs. In addition to the conjugative transfer system, *O*. *tsutsugamushi* retains a relatively large repertoire of genes for recombination and repair processes, which may ensure genomic flexibility during recurrent host changes and induce genetic variation, especially in surface antigens for immune evasion [45,66]. Considering that *tsa56* encodes the major outer membrane protein that plays a significant role in bacterial invasion into host cells [67,68] and the neutralizing antibodies against it provide protection [69], immunological pressure on the bacterial antigen during mammalian host infection might be a crucial driver for genetic diversification via point mutations, Indels, and genetic exchange among different genotypes. Indeed, our current study revealed that the mutation rate of *tsa56* gene is substantially higher than other house-keeping genes (Fig 6). This higher mutation rate was also observed in a group of outer membrane proteins encoded by *sca* family genes [50,70,71]. It is notable that the mutation rate of the *scaA* gene, which encodes a potential bacterial adhesin [72], is the second highest compared to other included genes. In addition to the point mutations, intragenic recombination may also contribute to the genetic diversity of the outer membrane proteins. Even though the recombination rate of the *tsa56* gene is similar to those of other *Orientia* genes (Fig 6), intragenic shuffling of the gene fragments has significantly contributed to the genotype diversification (Fig 4). Given that the extracellular domain of TSA56 includes highly variable domains [22], as well as multiple antigenic domains [73], intragenic recombination observed among the diverse genotype sequences can result in substantial shifts in genetic variation [27] and antigenicity [74]. In consistent with these, similar result was reported from a study using multiple *tsa56* sequences isolated from human patients from three countries in Southeast Asia [75]. They also suggests that weak divergence in the core genome and ancestral haplotypes are maintained by permanent recombination in mites while the *tsa56* gene is diverging in higher speed potentially due to selection by the mammalian immune system [75]. Due to the wide antigenic variation, immunity generated by vaccine trials, or even after natural infection, do not provide effective cross-reactivity among numerous genotypes, and reinfection with scrub typhus is relatively common in highly endemic areas [76]. Various studies also showed inter-genotype variation in virulence in humans and rodents, ranging from unapparent disease to consistent fatal infection when untreated [1]. Considering that each genotype generation (Fig 5) classified based on the sequential recombination events in this study includes both virulent and avirulent genotypes and the relative virulence in challenged animals appears to be highly mouse-strain specific [77,78], the genetic recombination of *tsa56* gene may not be specifically associated with virulence for individual genotypes although detailed analysis on the degree of virulence need to be examined.

Classification of *O*. *tsutsugamushi* genotypes has been primarily based on the sequence variation of *tsa56* since it is unique to *O*. *tsutsugamushi* and highly variable in amino acid content due to multiple variable domains [1]. By the end of 2015, more than a thousand *tsa56* sequences (size range, ~ 150 to > 2,000 bases) were deposited in international sequence databases. Most sequences include the variable portions of the gene and were annotated as a specific strain name and/or genotype name based on sequence analysis. Our current results using sequences covering a complete or nearly complete coding sequence (covering at least 85% of the open reading frame, ≥ 1,251 bases corresponding to 417 amino acids) showed that genetic recombination might have occurred at multiple sites within the coding region. Therefore, genotypic classification using a small fragment, including only parts of variable domains, may not be sufficient to completely identify inter-strain variation within the target gene. Sequence analysis using the complete or nearly complete sequences including all the variable domains, as well as the region beyond the recombination break points near the 3’ and 5’ ends (Fig 4 and S6 Table) of the *tsa56* gene might be required to clearly define *O*. *tsutsugamushi* genotypes. In a previous study, Kelly *et al*. reported that *O*. *tsutsugamushi* genotypes can be classified into at least 9 definable clusters when they analyzed 135 complete or nearly complete (> 1,200 bases) *tsa56* genes [1]. Here, we identified at least 17 clusters of genotypes, belonging to 5 definable groups, when using 206 complete or nearly complete sequences (≥ 1,251 bases). Based on our sequence analysis, similarity and identity in amino acids also need to be considered to define the genotypes or groups since some genotypes, such as Gilliam and TA763 members, show unexpected higher similarity in amino acid sequences with phylogenetically distant genotypes (Fig 3 and S5 Table). As the number of *tsa56* sequences increases, the genetic variation is expected to further diversify when considering the high mutation rate and on-going recombination within the *tsa56* gene.

Currently, the geographical distribution of *O*. *tsutsugamushi* genotypes is a critical issue for the development of effective diagnostics and vaccine [2]. Antigenic variation generated by genetic diversification of the immunogenic major outer membrane protein, TSA56, complicates diagnosis and efforts towards vaccine development. Therefore, an investigation of genotype diversity and prevalence in local endemic areas needs to be continued not only for the epidemiological monitoring of scrub typhus, but also for the improvement of diagnostic accuracy and vaccine development [47,74]. In this study, we examined the distribution of *O*. *tsutsugamushi* genotypes using the sequence data and the related geographical information. In addition, we also reviewed spatiotemporal changes of the primary vector species to assess the association with epidemiological changes of scrub typhus. Based on extensive data analyses, we found some compelling epidemiological features of scrub typhus. First, the prevalence of diverse genotypes of *O*. *tsutsugamushi* and multiple vector species in Taiwan is quite marked when compared to those of other endemic countries. The local prevalence of *Leptotrombidium* species is generally determined by multiple environmental factors such as temperature, precipitation, and host diversity [79,80]. Considering that *L*. *deliense* and *L*. *scutellare* are the major vectors of scrub typhus in the southern tropical area and northern temperate region, respectively (Fig 8), the presence of both mite species as dominant vectors might be a good indicator of vector diversity. The subtropical climate of Taiwan, as well as its location in the center of the endemic area, might provide a natural environment for such a vector diversity. Although *L*. *deliense* is a major vector throughout the islands of Taiwan, *L*. *imphalum* and *L*. *pallidum*, which are also primarily found in tropical area and temperate region, respectively, were more dominant in some Taiwanese provinces [80]. In addition, *L*. *deliense* was replaced by *L*. *scutellare* during the winter season in islands with lower winter temperature than the other areas, such that the former is responsible for summer scrub typhus and the latter for winter scrub typhus [80]. It is also interesting to note that the recent exponential increase of scrub typhus cases in mainland China has been primarily associated with regional clusters of the southern subtropical area [9,57], which is geographically close to Taiwan. Recently, the presence of highly diverse mite species was reported in the Yunnan province, the main hotspot of scrub typhus in mainland China [9], where *L*. *scutellare* and *L*. *deliense* are the major mite species [79,81]. The dominance of the two major vectors, as well as species diversity, are associated with local altitude and latitude gradients, suggesting an importance of climate and environmental conditions for codominance of mite species [79]. Although the genotype diversity of *O*. *tsutsugamushi* in endemic hotspots of southern China has been poorly defined, fluctuation and variety of the vector species due to environmental factors could also be associated with epidemiological features of scrub typhus in local endemic regions. Additionally, local changes in prevalent mite species harboring *O*. *tsutsugamushi* have been continuously reported in other subtropical and temperate area of endemic regions [16,80]. Ecological changes in the specific endemic locality may provide the environmental basis for the diversification of *O*. *tsutsugamushi* genotypes and/or their prevalence.

Second, presence of identical or near-identical (1 ~ 2 different bases) *tsa56* genes in geographically distant countries suggests a potential of international migration of *O*. *tsutsugamushi*, even though the genomic identity of the clones needs to be further verified. Considering that *O*. *tsutusgamushi* are obligate intracellular bacteria, their migration is absolutely dependent on the associated host vectors and/or reservoir animals. Moreover, larval mites do not migrate more than a few meters from where they hatch and usually form ‘mite islands’ ranging from a few cm to meters [19], so their ability to migrate on their own is very limited and their movement is mainly associated with the migration of hosts infested with chigger mites [3]. In addition to the wide spread of major vectors and diverse genotypes of *O*. *tsutsugamushi* over the endemic region including many islands in the Indian and Pacific Oceans [1], continuous fluctuation in the distribution of chigger mites at the local level suggests that parasitized small rodents and birds may be potential phoretic hosts of the infected mites [3,19,82,83]. A recent study reported a potential role of an exotic rodent species introduced from Southeast Asia and Pacific islands, *Rattus exulans*, as a host for chiggers in Taiwan [83]. Even though exotic *R*. *exulans* appears to play a relatively minor role in supporting chigger species infected with *O*. *tsutsugamushi* in Taiwan, the fact that both prevalence and loads of chiggers in *R*. *exulans* vary greatly with environment, along with the abundance and the ecological flexibility of *R*. *exulans*, implies a potential health risk as this species expands to areas with more chiggers [83]. Since the role and influence of exotic rodent species in local diversity and spread of the vector-borne disease are important but poorly assessed thus far, further investigation on the role of invasive rodent hosts on the dynamics of scrub typhus needs to be followed. Additionally, the association of migratory birds in spreading vector-borne infectious agents, such as *Borrelia burgdorferi* [84], Tick-borne encephalitis virus [85], and severe fever with thrombocytopenia syndrome virus [86], has been well documented. Considering that chigger mites attach and feed on host animals, including birds and rodents, for about 36–72 hours and withstand harsh environmental condition such as temperatures of -20°C for up to several weeks [87], they can travel hundreds to thousands of kilometers while attached to migratory birds, to a new geographic area that they may colonize if environmental conditions are optimal for their survival [88]. Since *O*. *tsutsugamushi* has rarely been recovered from tissues of wild birds [19,89], birds are more likely mechanical carriers for short- or long-distance transmission of chigger mites infected with *O*. *tsutsugamushi* rather than biological carriers. Based on our examination of the distribution of identical or near-identical *tsa56* sequences, Taiwan was found to be the nodal point of clonal expansion to northern and southern parts of the endemic area (Fig 9A). This further supports the idea that Taiwan, located in the center of the endemic area, may serve as a hub point mediating potential migration or expansion of vector mites, thereby enabling the generation and/or spread of diverse genotypes. Taiwan is also located at the center of the East Asia/Australasia Flyway migratory bird routes crossing the endemic countries of scrub typhus, extending from Arctic Russia to the southern limits of Australia (Fig 9B). In addition, the major habitats of the migratory bird species appear to be correlated with the endemic area of scrub typhus. Therefore, consideration of avian migration patterns might be useful in understanding and predicting epidemiological changes, such as local outbreaks of scrub typhus, as well as spread of mite vectors and *O*. *tsutsugamushi* genotypes. Fluctuation and diversification of vector species harboring *O*. *tsutsugamushi*, potentially caused by environmental changes and influx from other endemic regions, could affect the epidemiological features of scrub typhus and facilitate the genetic recombination among the different genotypes, thereby enhancing the genotypic diversity of *O*. *tsutsugamushi* in local endemic regions. Careful monitoring of dominant mite species and the prevalence of *O*. *tsutsugamushi* genotypes associated with the vectors might be required to reveal the correlation of genotype diversification of *O*. *tsutsugamushi* with ecological vector changes.

## Supporting information

S1 Table Resources and references used to compile recent epidemiological data of scrub typhus. (XLS)

S2 Table List of 324 *tsa56* genes and their information used in this study. (XLS)

S3 Table Spatiotemporal information of nine mite species reported in endemic countries of scrub typhus. (XLS)

S4 Table Summary of genotype-specific InDels detected in 206 *tsa56* sequences. (XLS)

S5 Table Pairwise similarity and identity of 206 amino acid sequences of *tsa56* genes. (XLS)

S6 Table Summary of significant recombination events detected by recombination detection programs embedded in RDP suite. (XLS)

S7 Table Summary of recombination, mutation, and substitution rates of 53 *O*. *tsutsugamushi* gene sets. (XLS)

S8 Table List of identical and sub-identical sequence pairs reported in geographically distant countries. (XLS)
